# Intravitreal Bevacizumab in the treatment of neovascular glaucoma secondary to central retinal vein occlusion: a case report

**DOI:** 10.1186/1757-1626-2-176

**Published:** 2009-10-30

**Authors:** Tarek Alasil, Michael E Rauser

**Affiliations:** 1 Loma Linda University, Department of Ophthalmology, Loma Linda, California 92354, USA

## Abstract

**Introduction:**

Every eye with central retinal vein occlusion (CRVO) is at risk for developing neovascular glaucoma (NVG). Vascular endothelial growth factor (VEGF) has been shown to play a key role in the development of NVG in CRVO. Bevacizumab (Avastin; Genentech, San Francisco, CA) is a recombinant monoclonal antibody binding all isoforms of VEGF. Several studies have demonstrated intravitreal bevacizumab-induced regression of iris and angle neovascularisation associated with NVG.

**Case presentation:**

A 74 year old female presented with acute onset decreased vision in the right eye. Ophthalmic exam revealed acute non-ischemic CRVO in the right eye. A month later, follow up exam showed progression into ischemic CRVO and secondary NVG, which was successfully treated with intravitreal Bevacizumab followed by pan retinal photocoagulation (PRP).

**Conclusion:**

Our case report highlights the use of intravitreal Bevacizumab in combination with PRP for the treatment of NVG secondary to CRVO.

## Introduction

Retinal vein occlusion (RVO) is the most common retinal vascular disease after diabetic retinopathy with a cumulative 10-year incidence of 1.6% [1]. Central retinal vein occlusion (CRVO) is a disease of the elderly patients (age >50 years old). Major risk factors are hypertension, diabetes, and atherosclerosis. Other risk factors are glaucoma, syphilis, sarcoidosis, vasculitis, increased intraorbital or intraocular pressure, hyphema, hyperviscosity syndromes (polycythemia, multiple myeloma, Waldenstrom's macroglobulinemia, and leukemia), high homocysteine levels, sickle cell, and HIV [2].

Every eye with CRVO is at risk for developing neovascular glaucoma (NVG). Lowering intraocular pressure helps to improve retinal circulation in an eye with CRVO [3], and there is a 10% risk for development of RVO in the fellow eye [4].

Risk factors for developing neovascular iris in patients with CRVO are the amount of nonperfused retina, extent of retinal hemorrhages, male sex, and central vein occlusion of less than one month duration [5]. Visual acuity in patients with CRVO at baseline is a strong predictor for the development of iris and angle neovascularisation, as is the amount of nonperfusion seen by fluorescein angiogram [6].

Established modalities for the treatment of NVG include panretinal photocoagulation (PRP) to reduce the production of vasoproliferative factors by ischemic retina, and medical and surgical control of elevated intraocular pressure. Bevacizumab (Avastin; Genentech, San Francisco, CA) is a recombinant monoclonal antibody binding all isoforms of vascular endothelial growth factor (VEGF). Several studies have demonstrated intravitreal bevacizumab-induced regression of iris and angle neovascularisation associated with NVG with promising results [7-14].

We present a patient with history of age-related macular degeneration (AMD) and polycythemia vera who developed CRVO, which subsequently got complicated by NVG, and was successfully treated with intravitreal injection of Bevacizumab followed by PRP.

## Case presentation

A 74 year old Caucasian female presented to the Eye clinic complaining of blurry vision in the right eye that started one week prior to presentation.

Past medical history is significant for hypertension, dyslipidemia, diabetes, and polycythemia vera. Past ocular history is significant for age related macular degeneration (AMD), cataract extraction and intraocular lens placement in both eyes. Most recent lab check showed hemoglobin of 16.5 gram per deciliter, and Hematocrit of 48.8%.

**Figure 1:**
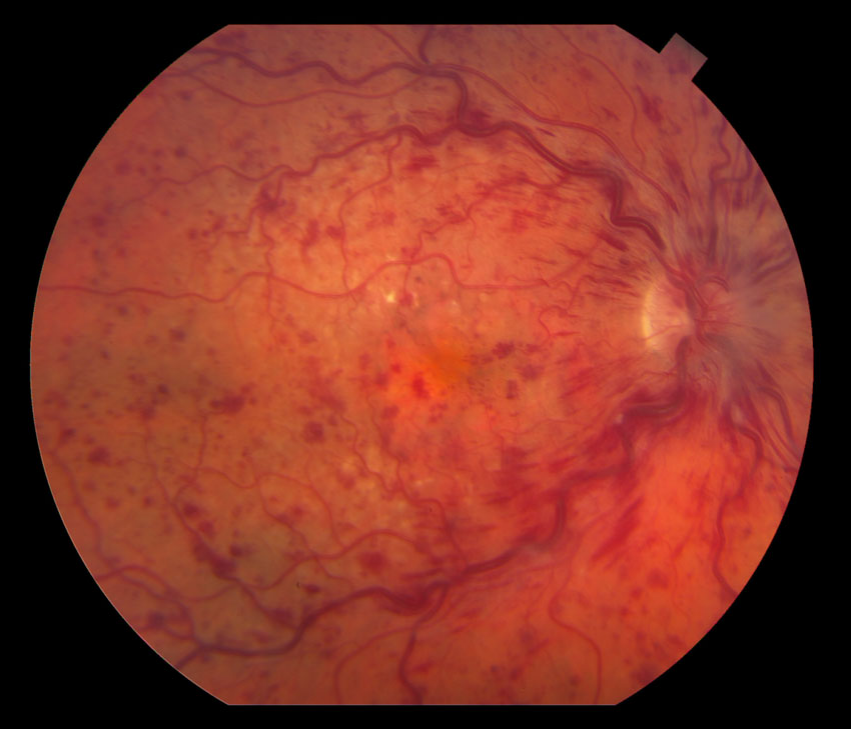
**Color fundus photograph of the right eye is showing the presence of venous tortuosity with distension**. There are moderate intraretinal hemorrhages throughout the posterior pole with a greater amount of hemorrhages in a peripapillary region. Mild disk edema appears to be present.

Ophthalmic exam revealed best corrected visual acuity of 20/CF in the right eye (OD), and 20/50 in the left eye (OS) [Baseline visual acuity was 20/30 OD, and 20/50 OS]. Pupil exam showed fixed irregular non-reactive right pupil that measured 4 mm. Left pupil was round, and reactive to light. Intraocular pressure measured 17 mm Hg on the right, and 14 mm Hg on the left. Slit-lamp exam showed normal lid and lashes, white conjunctiva, clear cornea, clear and deep anterior chamber bilaterally, and pseudophakia bilaterally (posterior capsule intraocular lens). Dilated fundus exam of the right eye showed mild disk edema, the presence of venous tortuosity with distension. There were moderate intraretinal hemorrhages throughout the posterior pole with a greater amount of hemorrhages in the peripapillary region (Figure 1).

**Figure 2:**
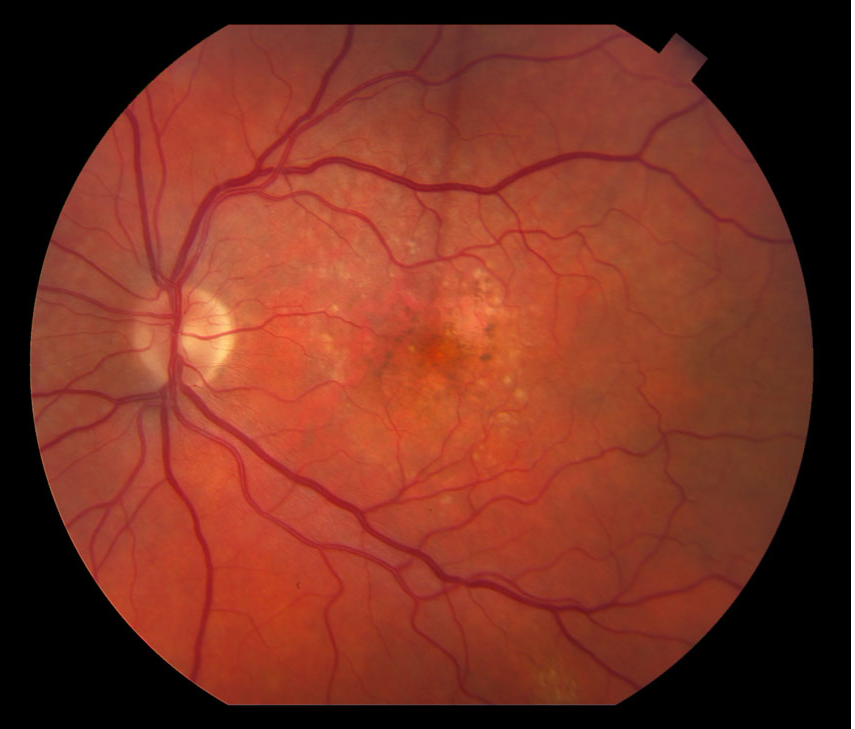
**Color fundus photograph of the left eye is showing the presence of drusen with RPE changes and focal areas of geographic atrophy in the macula consistent with macular degeneration**. Cup to disk ratio is 0.3 in the left eye with normal disk and vessels.

Dilated fundus exam of the left eye showed normal cup to disc ratio with normal disc and vessels, the presence of drusen with RPE changes and focal areas of geographic atrophy in the macula consistent with macular degeneration (Figure 2). The patient declined fluroescein angiogram.

Based on the clinical history and ophthalmic exam, the patient was diagnosed with acute non-ischemic central retinal vein occlusion (CRVO) in the right eye. The patient was instructed to follow closely in the Eye clinic, to perform gonioscopy and undilated examination of the iris on each visit to rule out NVG and iris neovascularization.

Follow-up exam one month later revealed visual acuity of 20/CF with no improvement on the right, and 20/50 on the left. Introcular pressures were 25 mmHg on the right, and15 mmHg on the left. Gonioscopy revealed angle neovascularization in the right eye. Dilated fundus exam was unchanged. The patient was diagnosed with NVG secondary to CRVO in the right eye.

She underwent an intravitreal Bevacizumab injection (1.25 mg/0.05 mL) and PRP in the right eye with an interval of one week to treat NVG. One week post PRP exam showed pressures of 18 mmHg OD and 15 mmHg OS.

## Discussion

CRVO is characterized by vascular obstruction leading to intraretinal hemorrhage, exudation of fluid, and variable degrees of ischemia. The vascular damage associated with the occlusion is accompanied by complex cellular and inflammatory reactions. A disturbed balance of angiogenic and inflammatory cytokines in ocular fluid has been observed in RVO [15-18].

VEGF is a known chemoattractant for macrophages and monocytes, a key factor in angiogenesis and increased vascular permeability. VEGF stimulates neovascularization and the formation of macular edema in CRVO [19]. Funk et al. found an increased VEGF levels in the aqueous humor of patients with CRVO. They also demonstrated measurable levels of VEGF in the control group providing evidence for a physiological expression of this angiogenic factor in the human eye [18]. They supposed that cytokine levels in aqueous humor should reflect levels in the vitreous, as correlation of aqueous and vitreous levels has been described in the past [20]. Additionally, there has been no correlation between cytokine levels in the aqueous humor and in plasma [15,20], providing evidence for intraocular sources.

VEGF mRNA up-regulation has been observed in the human retina in patients with CRVO [21] as has a correlation of the severity of macular edema with aqueous and vitreous levels of VEGF [20]. Funk et al. concluded that initial monthly treatment with anti-VEGF in CRVO reduced VEGF levels to undetectable values and below physiologic levels. The changes of intraocular VEGF levels were also associated with disease activity [18].

NVG secondary to CRVO is a devastating type of glaucoma with a poor prognosis. It is caused by neovascularisation of the iris and anterior chamber angle with eventual angle closure and intractable elevation of intraocular pressure (IOP) [14].

The management of NVG includes lowering IOP (often surgically) and PRP, which reduces the production of vasoproliferative factors by ischaemic retina and can induce regression of anterior segment neovascularisation.

Bevacizumab is a neutralizing anti-VEGF recombinant humanized monoclonal antibody. Intravitreal bevacizumab is now a frequently used adjunct for the treatment of VEGF-mediated NVG in CRVO [7-14]. Eyes treated with intravitreal bevacizumab should be monitored closely because surgery for IOP control and repeat bevacizumab injections are often necessary, regardless of initial angle status[14]. Bevacizumab should be instituted promptly after diagnosis, before irreversible anatomic and functional damage occurs [22].

Ehlers et al. compared combination intravitreal bevacizumab/PRP for the treatment of NVG with PRP alone. They concluded that the combination treatment resulted in more rapid decrease in IOP. In addition, the combination group had increased frequency and rapidity of regression of neovascularization [23].

Wakabayashi et al. studied 41 eyes with iris neovascularization (INV) or neovascular NVG secondary to ischemic retinal disorders. Patients received intravitreal Bevacizumab (1 mg) as the initial treatment for INV or NVG and were followed up for at least 6 months. Intravitreal bevacizumab was well tolerated, effectively stabilized INV activity, and controlled IOP in patients with INV alone and early-stage NVG without angle closure. In advanced NVG, Bevacizumab cannot control IOP but may be used adjunctively to improve subsequent surgical results [24].

## Conclusion

Our case illustrates an interesting presentation of CRVO in an old lady with multiple risk factors including polycythemia vera, hypertension, diabetes, and atherosclerosis. The patient progressed to an ischemic CRVO with secondary NVG. Her risk factors to develop NVG secondary to CRVO were the extent of retinal hemorrhages, CRVO of less than one month duration, and counting finger visual acuity upon initial presentation with CRVO. She was successfully treated with intravitreal Bevacizumab injection followed by PRP. The neovascularization regressed, and the intraocluar pressure was controlled.

In summary, our case report highlights the use of intravitreal Bevacizumab in combination with PRP in the treatment of NVG secondary to CRVO.

## Consent

Written informed consent was obtained from the patient for publication of this case report and accompanying images. A copy of the written consent is available for review by the Editor-in-Chief of this journal.

## Competing interests

The authors declare that they have no competing interests.

## Authors' contributions

*TA analyzed and interpreted the patient data, and wrote the manuscript.

- MR was the attending who evaluated the patient and performed the Bevacizumab intravitreal injection and the panretinal photocoagulation.
